# Special Issue: “Molecular Mechanisms and Regulation in Chronic Kidney Diseases”

**DOI:** 10.3390/ijms27031500

**Published:** 2026-02-03

**Authors:** Charlotte Delrue, Joris R. Delanghe, Marijn M. Speeckaert

**Affiliations:** 1 Department of Nephrology, Ghent University Hospital, 9000 Ghent, Belgium; charlotte.delrue@ugent.be; 2 Department of Diagnostic Sciences, Ghent University, 9000 Ghent, Belgium; joris.delanghe@ugent.be; 3 Research Foundation-Flanders (FWO), 1000 Brussels, Belgium

Chronic kidney disease (CKD) has become one of the most important medical challenges today, affecting approximately 850 million people worldwide [1,2,3]. Furthermore, CKD is responsible for a disproportionate morbidity (cardiovascular disease and frailty) and premature mortality compared with other diseases. Despite improvements in patient care and risk factor management, many patients with CKD still progress to kidney failure [3,4,5,6]. CKD is complex due to the interaction of molecular, metabolic, inflammatory, and genetic factors. The purpose of this Special Issue, titled “Molecular Mechanisms and Regulation of Chronic Kidney Diseases,” was to gather high-quality articles that explore the pathogenesis of CKD from intracellular signaling to systemic metabolic crosstalk and genetic susceptibility, while also identifying opportunities for the early identification and treatment of CKD. The articles published in this Special Issue demonstrate that nephrology is currently moving from a descriptive pathology approach to a more mechanistically based precision- or target-oriented approach.

In current CKD research, dysregulation of intracellular signaling pathways has been identified as a major contributor to renal cell fate, their ability to adapt to stress, and their capacity for maladaptive repair. A key example is the cyclic adenosine monophosphate (cAMP) pathway. Delrue et al. provided a comprehensive overview of how compartmentalized cAMP signaling regulates renal epithelial transport, mitochondrial dynamics, inflammatory signaling, and fibrotic responses [7]. The cAMP pathway is now recognized as an intricate signaling network that requires precise spatial organization to optimize biochemical effects on target cells. Otherwise, the activity of these signaling molecules may be significantly attenuated or functionally ineffective. Recent developments suggest that localized phosphodiesterase (PDE) activity and anchoring proteins (AKAPs) exist within each microdomain, determining cAMP distribution within the cell. These two factors work together to produce a range of downstream signaling effects that can lead to physiologically relevant changes through both protein kinase A (PKA) and Epac [8,9,10]. Emerging evidence suggests that disruption of the microdomain structure can lead to tubulointerstitial fibrosis, polycystic kidney disease, and reduced stress response capability in tubular epithelial cells [11,12]. The authors’ view reflects a general shift in our understanding of newer methodologies in signal transduction. It demonstrates that therapeutics acting on second messengers must be both spatially and pathway-specific to prevent possible adverse systemic effects, a consideration that is becoming increasingly relevant for CKD drug development [7].

Other ways to integrate CKD etiology with metabolism, mitochondria, and oxidative stress have been described. Reduced mitochondrial biogenesis, altered oxidative phosphorylation, and excess reactive oxygen species (ROS) production contribute to tubular injury, endothelial dysfunction, and fibrogenesis [13,14,15]. Pro-inflammatory signaling pathways have metabolic and mitochondrial stress as their components. Both the nuclear factor kappa-light-chain-enhancer of activated B cells (NF-κB) and the inflammasome pathways leading to interleukin-1β (IL-1β) production are tightly interconnected signaling cascades that form self-reinforcing inflammatory feedback loops that promote CKD. Even when the initial injury is resolved, maladaptive repair may create a continued state of inflammation [16,17,18,19,20]. In this context, the gut–kidney axis has emerged as a critical amplifier of metabolic and inflammatory stress. According to Młynarska et al. [21], many other studies have confirmed that changes to the microbial environment within the intestines of patients with CKD undergoing hemodialysis can cause an increase in the levels of many uremic toxins. High levels of the two forms of sulfonated indoxyl sulfate and p-cresyl sulfate activate the hydrocarbon receptor (AHR), which causes increased ROS and decreased function of tubular epithelial and endothelial cells in the kidney. This indicates that changes in the gut microbiome composition may directly impact the factors that contribute to kidney damage related to CKD [22]. Furthermore, Młynarska et al. [21] proposed various methods to modulate the gut microbiome using medications that enhance the bioavailability of dietary substrates and methods to improve the function of the intestinal barrier. Although significant barriers must still be overcome to implement such therapies in clinical practice, they may have favorable clinical outcomes when used in conjunction with nephroprotective therapies and may be important for the overall systemic regulation of the metabolism of patients with CKD.

A persistent limitation in CKD care is the reliance on late functional markers, including serum creatinine and albuminuria, which often increase only after substantial nephron loss [23]. Rhode et al. [24] closed this gap in the literature by creating urinary proteins that serve as a marker for “early stage” glomerular injury, before any evidence of albuminuria or inflammatory changes. These findings support ongoing efforts to create a new category of “mechanism-based” biomarkers that will enable the detection of reversible renal impairment. These methods have become increasingly parallel to the rapid expansion of proteomics, metabolomics, and single-cell transcriptomics, which have generated disease-specific molecular signatures before overt clinical manifestations of the disease become apparent [25]. The ability to detect diseases early is especially important for children and individuals with genetically inherited kidney diseases, as timely intervention can significantly affect their long-term health trajectory.

Diabetic nephropathy (DN) is a major cause of CKD driven by multiple converging pathogenic mechanisms, including oxidative stress, inflammation, and hemodynamic changes. As a result, if treatment only targets one type of injury, it will not be sufficient to treat patients with DN [26,27,28,29]. Morones-Gamboa et al. [30] demonstrated that combined pharmacological modulation using α-adrenergic antagonists, peroxisome proliferator-activated receptor (PPAR) agonists, and incretin-based pathways exerted additive renoprotective effects in a mouse model of DN. In addition, new clinical findings illustrate that combination treatment with sodium glucose cotransporter 2 (SGLT2) inhibitors combined with glucagon-like peptide (GLP-1) agonism offers additional renal and cardiovascular safety compared to each drug alone [31,32,33]. Aguilera-Martínez et al. [34] demonstrated the cellular mechanisms by which tamsulosin and pioglitazone inhibit hyperglycemia-mediated mesangial cell activation, oxidative stress, and extracellular matrix accumulation. Thus, these studies highlight drug repurposing and multi-pathway targeting as practical strategies for treating the complexity of CKD. A schematic representation of these interconnected mechanistic pathways is shown in Figure 1.

Genetic kidney diseases provide critical insights into the pathogenesis of CKD and illustrate the importance of molecular diagnosis. Cerkauskaite-Kerpauskiene et al. [35] characterized *COL4A3* and *COL4A4* variants in a Lithuanian cohort with Alport syndrome, revealing substantial genetic and phenotypic heterogeneity. Their findings reinforce emerging evidence that the Alport syndrome is more prevalent than previously appreciated and is often underdiagnosed due to variable clinical expression [36,37,38]. This study [35] exemplifies the growing impact of genomic medicine in nephrology. It uses a genetic diagnosis early in the treatment process to establish the prognosis, assist with follow-up decisions, aid with familial counseling, and ultimately provide therapies based on each patient’s genotype. This is an example of the transition to precision nephrology, in which the molecular cause of a disease determines how it is classified and treated, instead of relying solely on histopathologic patterns.

The collective contributions of this Special Issue support the view that CKD is a network condition developed by several factors working together: intracellular signaling dysregulation, metabolic and mitochondrial stress, immune activation, genetic susceptibility, and systemic crosstalk. Advances in the use of various omics technologies, experimental models, and systems biology for research and development have greatly enhanced our understanding of these networks and the ability to provide new diagnostic and treatment options beyond those currently used [39,40,41]. For the future, success will be built upon translating molecular knowledge into detection methods based on the molecular mechanisms of the condition, the identification of biomarkers for the condition, and the development of conventionally based precision treatment of the conditions. The studies discussed in this Special Issue add significant conceptual and experimental bases for this approach and demonstrate the value of interdisciplinary collaboration in advancing CKD-related research.

## Figures and Tables

**Figure 1:**
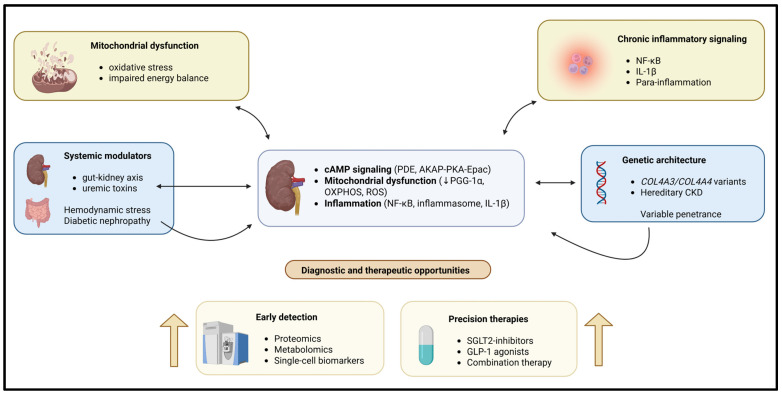
Chronic kidney disease (CKD) is caused by multiple interacting molecular and systemic mechanisms leading to CKD. Intracellular signaling is disrupted, causing the dysregulation of normal mitochondrial and metabolic functions. Chronic inflammation, genetic susceptibility, and environmental influences combine to produce pathological changes in the kidney. Pathways exist within the kidney cells that may form self-reinforcing feedback loops leading to CKD, resulting in increased renal injury and fibrosis. The development of omics-type diagnostic tools, as well as the development of combination therapies, creates excellent opportunities for the recognition and earliest use of precision treatment before irreversible deterioration of renal function occurs.

## Data Availability

Not applicable.

## Abbreviations

The following abbreviations are used in this manuscript:

AhR	Aryl hydrocarbon receptor
AKAP	A-kinase anchoring protein
AKI	Acute kidney injury
cAMP	Cyclic adenosine monophosphate
CKD	Chronic kidney disease
DN	Diabetic nephropathy
Epac	Exchange protein directly activated by cAMP
GLP-1	Glucagon-like peptide-1
IL-1β	Interleukin-1 beta
NF-κB	Nuclear factor kappa-light-chain-enhancer of activated B cells
PDE	Phosphodiesterase
PGC-1α	Peroxisome proliferator-activated receptor gamma coactivator 1 alpha
PKA	Protein kinase A
PPAR-γ	Peroxisome proliferator-activated receptor gamma
ROS	Reactive oxygen species
SGLT2	Sodium–glucose cotransporter 2
